# Nudging by design: calibrating climate and health interventions

**DOI:** 10.1016/j.joclim.2026.100724

**Published:** 2026-08-12

**Authors:** Morten Ploug Henriksen

**Affiliations:** University of Southern Denmark, Campusvej 55, Odense, Denmark

**Keywords:** The intervention ladder, Nudging, Climate change, Health, Policy, The nudge intensity model

## Abstract

**Introduction:**

Climate and health challenges increasingly require behaviorally informed approaches that complement structural policy tools. Nudging—changes in choice architecture designed to pull or push people in certain directions—has become widely used across climate and public health domains. However, its application varies considerably in both intensity and disciplinary orientation.

**Key findings:**

This article introduces the Nudge Intensity Model, a conceptual framework that positions nudges along a continuum from soft to moderate to hard interventions. Drawing on insights from communication, social and environmental psychology, design, policy studies, and economics, the model clarifies how different forms of nudging exert varying degrees of behavioral influence and how they can be calibrated to context, ethical considerations, and policy goals. Illustrative examples from climate and health interventions demonstrate the need for more deliberate calibration.

**Conclusion:**

The model aims to support practitioners in designing context-sensitive, legitimate, and effective behavioral interventions.

## 1 Introduction

Climate change and public health are increasingly interconnected, creating behavioral challenges that require coordinated and scalable solutions [1,2]. Rising temperatures, pollution, and shifting disease patterns place pressure on health systems while simultaneously demanding rapid reductions in carbon emissions. Traditional regulatory and infrastructural policies remain essential; however they often encounter political resistance or public reactance when perceived as overly restrictive [3]. Behavioral science offers complementary approaches that can promote climate-friendly and health-enhancing actions while preserving individual autonomy.

Among these approaches, nudging—changes in choice architecture designed to pull or push people in a certain direction—has gained prominence across research fields [4,5]. Nudges draw on well-established insights into cognitive biases, habits, and social norms [6,7] and have been applied to domains such as energy conservation [8], sustainable food choices [9], physical activity [10], and risk reduction [11]. A central normative rationale behind many nudges is libertarian paternalism, which promotes the idea that people should have some kind of freedom of choice while allowing for the possibility of steering them toward a certain behavior. While nudges can be effective, their ethical legitimacy and practical impact depend heavily on how strongly they steer behavior and how transparently nudges are implemented [12,13]. Debates about manipulation, autonomy, and proportionality highlight the need for clearer ways to differentiate and justify various forms of nudging [14,15].

The Nuffield Intervention Ladder provides a useful backdrop by categorizing interventions according to their intrusiveness, ranging from monitoring behavior and providing information to restricting or eliminating choice [16,17]. Although nudges are often placed on the middle rungs, practitioners across disciplines apply nudging techniques with varying degrees of behavioral force. The inclusion of extreme forms of interventions – such as eliminating choice altogether and monitoring – serves here as an analytical reference point rather than as a claim that such measures constitute nudges. These boundary cases help clarify how more or less forceful forms of choice architecture relate to one another and where nudging interventions are situated relative to other tools. This variation underscores the need for a more nuanced framework that can help policymakers and designers calibrate nudges to context, goals, and ethical considerations.

This article introduces the Nudge Intensity Model, a conceptual tool that positions nudges along a spectrum from soft to moderate to hard. The model aims to support more deliberate, transparent, and context-sensitive design of behavioral interventions in climate and health policy.

## 2 Conceptual approach

This article adopts a conceptual approach that synthesizes insights from communication, social and environmental psychology, design, policy studies and economics [6,18,19]. The aim is to integrate influential ideas and illustrative examples to clarify how nudging interventions vary in their behavioral force and how this variation can be calibrated in practice. Here, calibration refers to the deliberate alignment of an intervention´s intensity with the specific decision context, the behavioral barriers it seeks to address, and normative expectations regarding acceptable forms of public intervention.

The Nuffield Intervention Ladder serves as an ethical and structural backdrop [16], but existing applications of nudging often cut across its categories in different ways. Rather than treating nudges as a uniform category, the article emphasizes recurring patterns in how interventions differ in the degree to which they structure choice, alter incentives, or constrain behavior.

By drawing on interdisciplinary perspectives, the article identifies recurring patterns in how nudges are designed and justified, using these patterns to motivate the development of the Nudge Intensity Model. This conceptual synthesis supports a more coherent vocabulary for comparing nudges and offers practitioners a practical tool for aligning intervention design with context, acceptability, and policy goals.

## 3 Variation in nudging intensity

Behavioral interventions vary in how strongly they steer behavior. At the "hard" end of the spectrum are regulatory measures that eliminate or sharply restrict options. Denmark’s trans-fat ban improved cardiovascular outcomes [20], and the EU’s planned phase-out of fossil-fuel vehicles from 2035 reflects similar logic of restructuring choice environments to reduce harmful defaults, supported by a relatively high level of public preferences for regulatory phase-outs in this domain [21]. Different kind of smoking bans likewise reduced exposure to tobacco and contributed to declines in smoking rates [22]. Such measures are effective but raise concerns about autonomy and legal constraints [4,23]. Less intrusive restrictions increase friction or limit access. Carbon taxes reduce high-emission behaviors [[24], [25], [26]], and advertising bans or spatial restrictions reduce exposure to unhealthy products [27]. Bonus systems shift transport preferences [28,29], and rebates promote healthier consumption [30,31]. These interventions include incentives and disincentives and reshape environments without fully removing choice.

Moderate nudges include defaults interventions that steer behavior be pre-selecting an option while preserving the possibility of opting out. Automatic enrollment in green energy contracts increases renewable uptake [32], and plant-based meal defaults boost vegetarian choices [33,34]. These interventions are more forceful than simple changes in salience because they leverage status quo bias, and their effectiveness depends on legitimacy and transparent opt-out options [35,36].

Softer nudges enable choice by making climate-friendly or healthy options more visible or convenient. Altering availability and positioning can shift food choices with minimal effort [18], and menu design – through ordering, labeling, or visual emphasis – can encourage more responsible consumption [37,38]. Informational interventions raise awareness while preserving autonomy. CO₂ labels and sustainability campaigns increase visibility of environmental impacts [[39], [40], [41]], though their influence is often limited by habits and context [42]. Monitoring itself can act as a subtle nudge. This is illustrated by the Hawthorne effect [43,44], where people adjust their behavior simply because they know they are being observed or measured. Tools such as Life Cycle Analysis databases make environmental trade-offs visible and can steer choices without overt regulation [45].

These variations reflect deeper disciplinary orientations. Communication research tends to emphasize information and framing [46,47]; behavioral economics focuses on incentives and default rules [4]; design fields work through spatial and material arrangements [[48], [49], [50]], and public health traditions often prioritize regulation, equity, and structural determinants [11]. As a result, interventions labeled as “nudges” can differ substantially in their behavioral force, ethical implications, and expected impact.

This variation makes it difficult for practitioners to compare interventions across sectors and contexts. It underscores the need for a more systematic way to understand and calibrate nudges so that interventions can be tailored to local conditions, policy goals, and public legitimacy.

## 4 The nudge intensity model

The Nudge Intensity Model offers a structured way to distinguish soft, moderate, and hard nudges along a continuum of behavioral influence. Rather than treating nudging as a single, uniform category, the model clarifies how interventions differ in the degree to which they steer choices and how these differences relate to disciplinary research traditions. The two extremes – do nothing, and eliminate choice – do not constitute nudges as such but serve as analytical boundary points that define the outer limits of behavioral influence against which nudging interventions can be calibrated.

**Figure:**
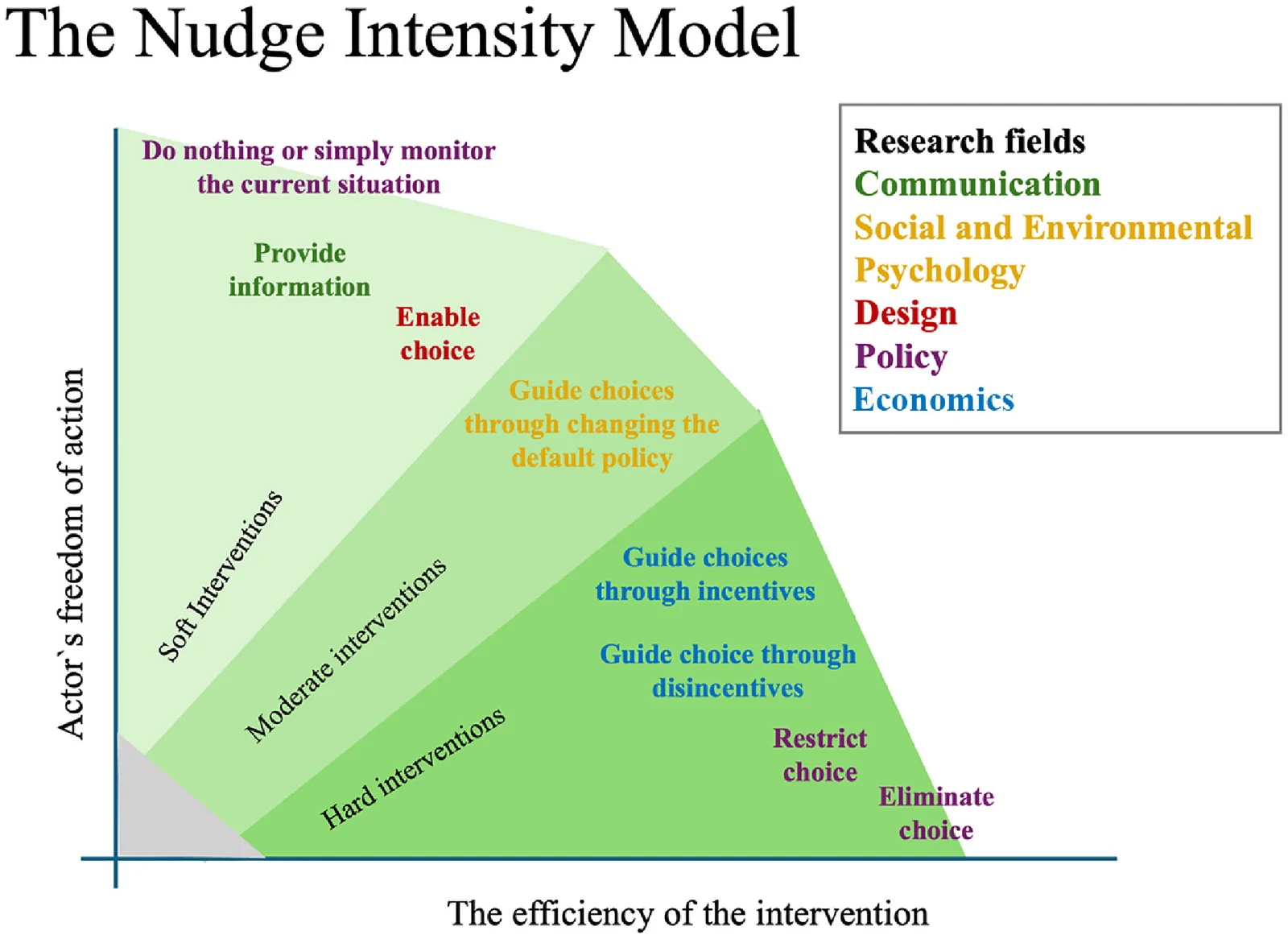

Soft nudges rely on informational cues and subtle framing to raise awareness while preserving maximal autonomy [9]. Moderate nudges reshape the choice environment through defaults, salience, and environmental design, making certain actions easier or more likely without restricting alternatives [18,51]. Hard nudges introduce friction or constraints that make some behaviors less convenient or less prominent, while still maintaining the possibility of opting out [28,33]. These distinctions help clarify why interventions with similar goals can differ substantially in public acceptability, ethical justification, and behavioral impact.

A central premise of the model is that no single level of intensity is inherently superior. Effective intervention design requires situational sensitivity: softer nudges may be appropriate when legitimacy, autonomy and low resistance are paramount [13,52], whereas moderate or harder nudges may be necessary when behaviors are habitual, environmentally harmful, or resistant to change [40,53]. Harder interventions introduce friction or constraints and can, in some cases, produce stronger or more durable behavioral effect, but they also carry a greater risk of eliciting pushback or reducing perceived freedom [35,54]. The model also underlines opportunities to combine nudges across the spectrum, enabling softer and harder elements to reinforce one another in climate and health interventions. Such combinations can involve pairing low-resistance informational cues with more forceful environmental or structural changes, creating interventions that both support autonomy and achieve meaningful behavioral impact.

For example, reducing car-related air pollution in urban areas can involve interventions across the intensity spectrum. Soft nudging includes information campaigns and CO₂ labels that raise awareness and promote alternative transport. Moderate nudges restructure the choice environment through defaults and infrastructure design, such as prioritizing bike lanes or improving public transport convenience. Harder nudges involve congestion charges or low-emission zones and introducing stronger incentives or constraints while still allowing some choice. Which combination is appropriate depends on baseline behavior, public acceptance, and the urgency of climate and health risks.

Designing effective nudging interventions requires careful attention to public acceptance, perceived autonomy, and the broader governance context in which behavioral tools are deployed. People are more willing to accept nudges when they feel their freedom is preserved, when the intervention aligns with shared values, and when the underlying goals are transparent and trustworthy [[55], [56], [57]]. Conversely, interventions perceived as covert, overly directive, or misaligned with personal priorities risk triggering psychological reactance [3] and undermining policy support. This perspective supports a more deliberate approach to behavioral policy, one that acknowledges ethical tradeoffs while seeking to maximize both effectiveness and public legitimacy.

By positioning potential interventions along the intensity continuum, policymakers and researchers can quickly generate alternative versions of the same nudge and adapt them to different contexts, constraints, and target groups. Building on this, future research should investigate how interventions across the spectrum from soft to hard nudges can be strategically combined to reinforce one another. It should also examine which interventions work best for personal behavioral change versus systemic change, and how impact can be scaled across intervention categories.

## 5 Conclusion

Nudging offers an important but underutilized opportunity for integrating behavioral insights into climate and health policy. As interventions increasingly span communication, social and environmental psychology, design, policy studies and economics, the need for a clearer vocabulary to distinguish how strongly different nudges steer behavior has become more pressing. This article has argued that nudges vary along a continuum of intensity and that their efficiency and legitimacy depend on how well this intensity is calibrated to context, behavioral barriers, and public expectations.

The Nudge Intensity Model contributes to this effort by positioning nudges along a spectrum ranging from soft to moderate to hard and by clarifying how disciplinary traditions shape the design and justification of these interventions. Rather than reinforcing a binary distinction between “light-touch” and “heavy-handed” approaches, the model supports a layered strategy in which informational, structural, and regulatory elements can be combined and adapted to local conditions.

## Declaration of generative AI and AI-assisted technologies in the writing process

During the preparation of this work the author(s) used Microsoft Copilot in order to do language correction to US English. After using this tool/service, the author(s) reviewed and edited the content as needed and take(s) full responsibility for the content of the publication.

## CRediT authorship contribution statement

**Morten Ploug Henriksen:** Writing – review & editing, Writing – original draft.

## Declaration of competing interest

The authors declare that they have no known competing financial interests or personal relationships that could have appeared to influence the work reported in this paper.
